# Commentary: The Vaginal and Urinary Microbiomes in Premenopausal Women With Interstitial Cystitis/Bladder Pain Syndrome as Compared to Unaffected Controls: A Pilot Cross-Sectional Study

**DOI:** 10.3389/fcimb.2020.00064

**Published:** 2020-02-25

**Authors:** Magdalena Emilia Grzybowska

**Affiliations:** Department of Gynecology, Gynecological Oncology and Gynecological Endocrinology, Medical University of Gdansk, Gdańsk, Poland

**Keywords:** microbiome, PISQ-IR, sexual function, scoring, interstital cystitis, ICBPS

In this article, Meriwether et al. have assessed the vaginal and urinary microbiome to detect alterations associated with the presence of Interstitial cystitis/bladder pain syndrome (ICBPS). The study group included 23 women with ICBPS and 18 controls. No significant differences were found in the vaginal or urinary microbiome between the groups. ICBPS women presented lower scores in the applied questionnaires: Overactive Bladder Questionnaire, Pelvic Floor Distress Inventory-20, modified Body Image Scale and several Pelvic Organ Prolapse/Incontinence Sexual Questionnaire – IUGA Revised (PISQ-IR) domains in sexually active women. My main doubt concerns the PISQ-IR calculation system, which could influence the obtained results.

## Introduction

ICBPS is a disease of unknown etiology, with symptoms of intermittent or chronic pelvic pain and lower urinary tract symptoms such as frequency and urgency. Several therapeutic options are introduced, but none of them improves completely all the symptoms (Marinkovic, 2019). Thus, understanding the pathophysiology of ICBPS could be a milestone in the search for successful treatment. Recent studies focus on microbiome and its possible influence on different health conditions (Leprun and Clarke, 2019). Women with urgency urinary incontinence were found to have more bacteria and a more diverse urinary microbiome (Thomas-White et al., 2016). Another study suggests the protective role played by the more diverse, lactobacillus-dominated urinary microbiome among controls compared to women with interstitial cystitis (Abernethy et al., 2017).

Therefore it was very interesting to read this study combining the analysis of the urinary and vaginal microbiome (Meriwether et al., 2019). During enrollment patients were thoroughly screened for interfering conditions such as menopausal status, pelvic organ prolapse beyond the hymen, foreign bodies in the vagina, active urinary or genital tract infection and current treatment for ICBPS. The patients were administered several questionnaires: symptom assessing, quality of life, and sexual function, i.e., PISQ-IR.

## PISQ-IR

My concern is targeted on the PISQ-IR scoring system. PISQ-IR is a disease-specific questionnaire to assess sexual function in women with pelvic floor disorders (Rockwood et al., 2013). It incorporates questions for sexually active and inactive women. The sub-scales are calculated separately. The part of the questionnaire for sexually active patients contains the following six domains: Arousal/Orgasm, Condition Specific, Partner-related, Desire, Condition Impact, and Global Quality. It is also possible to calculate the Single Summary Score for sexually active patients (Constantine et al., 2017). The domains can be scored using either mean calculation or a transformed summation. Three methods of scoring PISQ-IR domains were evaluated by the authors of the questionnaire: simple summation, transformed summation, and mean calculation. Simple summation which is the process of just adding up the answers is not recommended. In order for simple summation to be accurate one of two conditions has to be met: all items have to be answered or imputation has to be used for unanswered items (Rockwood et al., 2013). This system can lead to significant error in scale calculation. Therefore, in order to help researchers, the scoring program—excel file is available on International Urogynecological Association (IUGA) website https://www.iuga.org/images/content/pisq-ir-scoring-program-V1.xlsx. In this program, the results are presented both, as mean calculation and a transformed sum score, leaving the decision on the preferred scoring system to the investigators (but not as a simple summation).

In the commented study the authors presented the PISQ-IR results without any information on the scoring system. Based on the Table 1 it seems that the simple summation process was used in the domains. For instance, in the Condition Impact domain the authors presented the results as 12.36 ± 3.57, which is the simple summation. The mean calculation system would present the scores as a sum of points (including appropriate reverse responses) divided per number of items answered with range 1.0–4.0 for that domain (Grzybowska et al., 2019). In a transformed summation, the mean scores would be transformed to a 0–100 range in each domain. The requirement for a simple summation method is all items answered (Rockwood et al., 2013). In the study on German validation of PISQ-IR the item non-response rates ranged from 2 to 40% in sexually active women (Trutnovsky et al., 2016), which constitutes a considerable problem in self-administered questionnaires.

As discussed before simple summation it is not the recommended way of calculating the questionnaire results. It is important to use questionnaires as intended for. Presenting the results of questionnaire analysis should be uniform in order receive correct results and to compare study outcomes between different cohorts.

## Sexual Function

Although the study is mostly focused on microbiology analysis it incorporates sexual function assessment, which is the strength of the study, of a more holistic approach to the ICBPS. Sexual dysfunction in female patients with ICBPS consists of vulvodynia, dyspareunia during or after sexual intercourse, altered sexual desire and orgasm frequency and insufficient lubrication (Gupta et al., 2015). It leads to diminished sexual activity and inhibited sex life (Gardella et al., 2011). In a study by Gardella et al. Female Sexual Function Index (FSFI) score of ICBPS patients was found significantly lower than the controls (16.85 ± 8.73 vs. 27.34 ± 6.41, *p* < 0.0001). Also in a study by Abernethy et al. participants with interstitial cystitis had lower scores on the FSFI, indicating more sexual dysfunction compared with the control group [17.3 (IQR 9.58–25.65) vs. 28.4 (IQR 9.95–33.125), *p* = 0.05] (Abernethy et al., 2017). After treatment, the improvement in sexual function was correlated with symptom resolution (Tonyali and Yilmaz, 2017). In the commented study the patients in the ICBPS group scored lower in Arousal/Orgasm, Global Quality, and Condition Impact domains (*p* < 0.05). However, no correlation was found between the sexual function and the diversity of the vaginal and urinary samples.

## Conclusions

As to my knowledge it is the first study to analyze sexual function in women with ICBPS with this relatively new measure—the PISQ-IR. Although calculated with simple summation, the questionnaire allowed to present differences in sexual function between the study groups. It would be interesting to investigate the groups again with recommended calculation systems such as mean calculation or transformed summation.

## Author Contributions

MG analyzed the original paper and wrote the commentary.

### Conflict of Interest

The author declares that the research was conducted in the absence of any commercial or financial relationships that could be construed as a potential conflict of interest.
